# The associations of *APP* , *PSEN1* , and *PSEN2* genes with Alzheimer's disease: A large case–control study in Chinese population

**DOI:** 10.1111/cns.13987

**Published:** 2022-10-10

**Authors:** Xuewen Xiao, Hui Liu, Lu Zhou, Xixi Liu, Tianyan Xu, Yuan Zhu, Qijie Yang, Xiaoli Hao, Yingzi Liu, Weiwei Zhang, Yafang Zhou, Junling Wang, Jinchen Li, Bin Jiao, Lu Shen, Xinxin Liao

**Affiliations:** ^1^ Department of Neurology, Xiangya Hospital Central South University Changsha China; ^2^ Bioinformatics Center && National Clinical Research Center for Geriatric Disorders Central South University Changsha China; ^3^ Department of Geriatrics, Xiangya Hospital Central South University Changsha China; ^4^ Department of Radiology, Xiangya Hospital Central South University Changsha China; ^5^ Engineering Research Center of Hunan Province in Cognitive Impairment Disorders Central South University Changsha China; ^6^ Hunan International Scientific and Technological Cooperation Base of Neurodegenerative and Neurogenetic Diseases Changsha China; ^7^ Key Laboratory of Hunan Province in Neurodegenerative Disorders Central South University Changsha China; ^8^ Key Laboratory of Organ Injury, Aging and Regenerative Medicine of Hunan Province Changsha China

**Keywords:** Alzheimer's disease, APP, PSEN1, PSEN2, the Chinese population

## Abstract

**Aim:**

The associations of non‐pathogenic variants of *APP*, *PSEN1*, and *PSEN2* with Alzheimer's disease (AD) remain unclear. This study is aimed at determining the role of these variants in AD.

**Methods:**

Our study recruited 1154 AD patients and 2403 controls. *APP*, *PSEN1*, *PSEN2*, and *APOE* were sequenced using a targeted panel. Variants were classified into common or rare variants with the minor allele frequencies (MAF) cutoff of 0.01. Common variant (MAF≥0.01)‐based association test was performed by PLINK 1.9, and gene‐based (MAF <0.01) association analysis was conducted using Sequence Kernel Association Test‐Optimal (SKAT‐O test). Additionally, using PLINK 1.9, we performed AD endophenotypes association studies.

**Results:**

A common variant, *PSEN2* rs11405, was suggestively associated with AD risk (*p* = 1.08 × 10^−2^). The gene‐based association analysis revealed that the *APP* gene exhibited a significant association with AD (*p* = 1.43 × 10^−2^). In the AD endophenotypes association studies, *APP* rs459543 was nominally correlated with CSF Aβ42 level (*p* = 7.91 × 10^−3^).

**Conclusion:**

Our study indicated that non‐pathogenic variants in *PSEN2* and *APP* may be involved in AD pathogenesis in the Chinese population.

## Graphical abstract

In this study, we systematically explored the relationship between Alzheimer's disease (AD) and non‐pathogenic variants of *APP*, *PSEN1*, and *PSEN2* in a total of 3557 individuals in a Chinese population. The common variant association test revealed that *PSEN2* rs11405 were nominally associated with AD. Gene‐based association analysis indicated that non‐pathogenic variants in *APP* gene may contribute to the etiology of AD. Our study indicated that non‐pathogenic variants in *PSEN2* and *APP* may be involved in AD pathogenesis in the Chinese population.

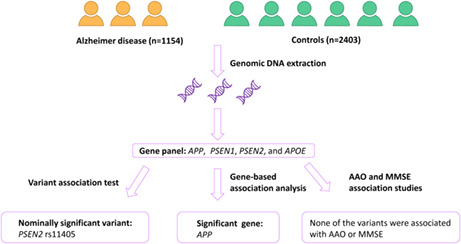

## 1 INTRODUCTION

Alzheimer's disease (AD) is a common progressive neurodegenerative disease characterized by memory decline and cognitive dysfunction. The prevalence of dementia is rising rapidly and imposing a heavy burden on families and societies.^1^ AD is a highly heritable disease with its heritability estimated to be as high as 60%–80%.^2^ The etiology of AD remains complex. Genetics plays an important role in AD development. Large‐scale genome‐wide association studies (GWAS) have identified 75 susceptibility loci in AD.^3^, ^4^, ^5^ However, the heritability of AD is still missing and remains to be identified.^6^ More genetic studies are required to illustrate the pathogenesis of AD.

Three genes, including amyloid precursor protein (*APP*), presenilin 1 (*PSEN1*), and presenilin 2 (*PSEN2*), are the causative genes of AD. The pathogenic variants of these three genes only presented a low proportion of AD patients (<5%). In our cohort, pathogenic/likely pathogenic variants were only identified in 2.2% of AD patients.^7^ Additionally, according to the American college of medical genetics and genomics and the association for molecular pathology (ACMG‐AMP) guidelines, 26.5% of variants of *APP*, *PSEN1*, and *PSEN2* were classified as variants of uncertain significance or benign variants (hereafter referred to as non‐pathogenic variants).^8^ A few studies examined the role of non‐pathogenic variants of *APP*, *PSEN1*, and *PSEN2* in AD pathogenesis. For example, in non‐Hispanic Caucasian individuals, the *PSEN1* p. E318G variant conferred susceptibility to AD only in patients carrying *APOE* ε4 allele.^9^

Nevertheless, the association of non‐pathogenic variants with AD received limited attention. Meanwhile, the sample size in some previous studies is limited.^10^ Consequently, to determine the role of non‐pathogenic variants of *APP*, *PSEN1*, and *PSEN2* in AD, we comprehensively analyzed these non‐pathogenic variants between AD patients and controls in a large Chinese population via a targeted sequencing panel.

## 2 METHODS

### 2.1 Participants

We recruited 1154 AD patients and 2403 controls from Xiangya Hospital and a community in Changsha. Based on the National Institute on Aging‐Alzheimer's Association criteria for probable AD^11^, the patients were diagnosed with AD by two expert neurologists. Participants with causative mutations for AD, vascular dementia, and frontotemporal dementia (including *C9orf72*) had been excluded by Sanger sequencing or repeat‐prime PCR (RP‐PCR) analysis. This study was approved by the Ethics Committee of Xiangya Hospital, Central South University, China. Written informed consent was obtained from each participant or guardian.

### 2.2 Genomic DNA isolation

Genomic DNA was extracted from the peripheral blood leukocytes using phenol‐chloroform extraction and ethanol precipitation.^12^ The DNA's quality and quantity were assessed via a NanoDrop spectrophotometer (Thermo Scientific). The DNA sample was diluted to 50–100 ng/μL.

### 2.3 Targeted gene sequencing

The targeted sequencing panel includes *APP*, *PSEN1*, *PSEN2*, and *APOE*. All of their exons and flanking regions were sequenced by our panel. The genomic DNA was broken into fragments with 150–200 bp length via Biorupter Pico, followed by end‐repairing, A‐tailing, adaptor ligation, and PCR amplification. Using the Illumina NovaSeq 6000 platform, the fragmented DNA was sequenced. The low‐quality reads fastq data were deleted by FastQC (http://www.bioinformatics.babraham.ac.uk/projects/fastqc/). The sequence reads were mapped to the human reference genome (UCSC hg19/GRCH37) using the BWA software (version 0.7.15, http://bio‐bwa.sourceforge.net).^13^ Duplicate sequence reads were removed by Picard (version 2.18.7, http://broadinstitute.github.io/picard/). The quality‐score recalibration, local realignments, and variant calling were performed by the Genome Analysis Toolkit (version 3.2, https://software.broadinstitute.org/gatk/).^14^ Variants were annotated using ANNOVAR (https://hpc.nih.gov/apps/ANNOVAR.html).^15^ According to minor allele frequencies (MAF), variants were classified into common or rare variants with the MAF cutoff of 0.01. In addition, we predicted the pathogenicity of missense variants by ReVe.^16^ In our study, the damaging variants included damaging missense variants (ReVe >0.7) or loss‐of‐function (LoF) variants. LoF variants involved nonsense, frameshift, or splicing variants.

### 2.4 Statistical analysis

The normality of data was tested using SPSS 26. Using PLINK 1.9,^17^ the variants with genotyping rate < 95%, genotype quality (GQ) ≤ 20, and Hardy–Weinberg equilibrium *p*‐value <1 × 10^−6^ in controls were excluded in our study. The common variant‐based association analysis was performed by PLINK 1.9. Age, *APOE* ε4 status (*APOE* ε4+, *APOE* ε4‐), and gender were adjusted for each common variant.

Furthermore, gene‐based association tests were performed by aggregating rare variants using the Sequence Kernel Association Test‐Optimal (SKAT‐O test).^18^ Rare variants were further divided into three groups: rare damaging variants (MAF <0.01, LoF or ReVe >0.7), rare damaging missense variants (MAF <0.01, ReVe >0.7), and rare missense variants (MAF <0.01, missense). Also, age, gender, and *APOE* ε4 status were also adjusted in the SKAT‐O test. According to Bonferroni correction, a cutoff *p*‐value <0.05/n was considered to reach statistical significance (n: the number of variants or genes).

## 3 RESULTS

### 3.1 Demographic and clinical information

1154 AD patients and 2403 controls were recruited in our study. The average age of onset of AD patients was 64.44 years old, and the average age of controls was 64.76 years old. No significant age difference was observed between AD patients and controls (*p* = 0.52). The AD patients' MMSE scores were lower than those of controls (*p* = 1.31 × 10^−12^) (Table 1).

**TABLE 1:** Demographic and clinical information of AD patients and controls

	AD	Control	*p*‐value
Number	1154	2403	–
Age(years), mean ± SD	64.44 ± 10.85	64.76 ± 7.78	0.52^a^
Gender(M/F)	458/696	1152/1251	4.37 × 10^−6^ ^b^
MMSE, mean ± SD	10.32 ± 7.61	26.75 ± 2.79	1.31 × 10^−12^ ^a^
MoCA, mean ± SD	8.23 ± 5.78	‐	‐
CDR, mean ± SD	1.29 ± 0.70	‐	‐
ADL, mean ± SD	33.58 ± 12.53	‐	‐
NPI, mean ± SD	17.41 ± 14.98	‐	‐

Abbreviations: AD, Alzheimer's disease; ADL, Activities of daily living; CDR, Clinical Dementia Rating; F, female; M, male; MMSE, Mini‐mental State Examination; MoCA, Montreal Cognitive Assessment; NPI, Neuropsychiatric Inventory; SD, standard deviation.

^a^ *p*‐value was calculated by Mann–Whitney *U* test (it did not exhibit a normal/Gaussian distribution).

^b^ *p*‐value was calculated by chi‐squared test.

### 3.2 Common variant association analysis

After quality control, seven common variants were observed in our study, including two *APP* variants, one *PSEN1* variant, and four *PSEN2* variants. These common variants were located in exons (42.9%, 3/7), introns (42.9%, 3/7), and 5′‐untranslated region (5’‐UTR; 14.2%, 1/7). The single common variant association test identified that a common variant in *PSEN2*, rs11405, was nominally linked to AD risk after adjusting for age, gender, and *APOE* ε4 status (*p* = 1.08 × 10^−2^) (Table 2). However, after the Bonferroni correction, this common variant was no longer associated with AD risk (*p* > 7.14 × 10^−3^).

**TABLE 2:** Common variants between AD patients and controls

Gene	Position	Rs ID	Region	Variant	Effect allele	MAF		OR (95% CI)	*p*	Adjusted *p*
						Case	Control			
*PSEN2*	1:227069677	rs11405	Exonic	c.69 T > C:p.A23A	T	0.452	0.482	0.875(0.801–0.979)	1.73 × 10^−2^	1.08 × 10^−2^
*PSEN2*	1:227071525	rs1046240	Exonic	c.261C > T:p.H87H	T	0.400	0.375	1.096(1.004–1.231)	4.23 × 10^−2^	8.73 × 10^−2^
*PSEN2*	1:227077809	rs75733498	Exonic	c.861C > T:p.P287P	T	0.076	0.074	1.074(0.851–1.241)	7.76 × 10^−1^	4.73 × 10^−1^
*APP*	21:27543049	rs459543	UTR5	–	G	0.182	0.186	0.965(0.853–1.109)	6.79 × 10^−1^	6.11 × 10^−1^
*PSEN1*	14:73664853	rs165932	Intronic	–	G	0.368	0.376	0.973(0.871–1.073)	5.23 × 10^−1^	6.15 × 10^−1^
*APP*	21:27254092	rs45513597	Intronic	–	G	0.030	0.027	0.998(0.824–1.490)	4.99 × 10^−1^	9.89 × 10^−1^
*PSEN2*	1:227058440	rs111297484	Intronic	–	G	0.000	0.038	NA(NA)	NA	NA

Abbreviations: AD, Alzheimer's disease; Adjusted P, adjusted by age, gender, and *APOE* ε4 status; CI, confidence interval; MAF, minor allele frequency; NA, not applicable; OR, odds ratio; Effect allele represents the minor allele.

### 3.3 Rare variant aggregation testing

We performed gene‐based aggregation testing by combing the rare variants within genes between AD patients and controls. In the rare missense variants group, after adjusting for age, gender, and *APOE* ε4 status, the *APP* gene exhibited a significant association with AD (*p* = 1.43 × 10^−2^) (Table 3). Specifically, 1.00% of the AD cases and only 0.29% of the controls carried *APP* missense variants. In the remaining three groups, including rare LoF variants, rare damaging missense variants, and rare damaging variants, none of these genes were associated with AD risk (Table S1–S9).

**TABLE 3:** Significant gene between AD patients and controls in the SKAT‐O test

Classification	Gene	Location	Variant	AD (*n*)	Control (*n*)
Rare missense variants (MAF <0.01)	*APP*	21:27277356	c.1943G > A:p.R648Q	1	0
		21:27284152	c.1810G > A:p.V604M	0	1
		21:27284214	c.1748A > G:p.E583G	0	1
		21:27327949	c.1579C > T:p.R527W	1	1
		21:27327979	c.1549A > C:p.M517L	1	2
		21:27328006	c.1522A > G:p.T508A	0	1
		21:27328065	c.1463G > A:p.R488H	1	0
		21:27347391	c.1450C > T:p.P484S	1	0
		21:27372339	c.1024G > A:p.G342S	5	0
		21:27372368	c.995A > G:p.D332G	2	0
		21:27372467	c.896C > G:p.P299R	1	0
		21:27372473	c.890C > T:p.T297M	6	2
		21:27394180	c.841G > C:p.E281Q	0	1
		21:27394215	c.806C > T:p.T269I	0	1
		21:27394275	c.746A > G:p.E249G	0	1
		21:27394327	c.694G > A:p.E232K	0	1
		21:27423503	c.475A > G:p.S159G	1	0
		21:27423508	c.470C > T:p.T157I	1	0
		21:27462283	c.331 T > C:p.F111L	0	1
		21:27484325	c.196A > G:p.K66E	1	0
		21:27484444	c.77C > G:p.A26G	1	0
		21:27542895	c.44C > T:p.A15V	0	1
Allele count/total number of alleles (n/n)				23/2308	14/4806
Frequency (%)				1.00	0.29
Adjusted P (SKAT‐O)				1.43 × 10^−2^	

Abbreviations: AD, Alzheimer's disease; Adjusted P, adjusted by age, gender, and *APOE* ε4 status; MAF, minor allele frequency; n, number; SKAT‐O, Sequence Kernel Association Test‐Optimal.

### 3.4 AD endophenotypes association studies

To illustrate the role of *APP*, *PSEN1*, and *PSEN2* in AD endophenotypes, we conducted AAO, MMSE, MoCA, CDR, and cerebrospinal fluid (CSF) biomarkers association studies in AD patients. In the AD endophenotypes association studies, seven common variants were left after quality control. After adjusting for gender and *APOE* ε4 status, none of these variants were linked to the AAO, MMSE, MoCA, and CDR in AD. In our study, a subgroup of 95 AD patients underwent CSF testing. CSF Aβ42, Aβ40, Total tau (T‐tau), and phosphorylated tau (P‐tau) were determined. In the CSF biomarkers association analyses, we identified that *APP* rs459543 was nominally correlated with CSF Aβ42 levels (*p* = 7.91 × 10^−3^) (Table 4). After the Bonferroni correction, rs459543 was no longer associated with CSF Aβ42.

**TABLE 4:** CSF Aβ42 association analyses in AD patients

Gene	Position	Rs ID	Region	Variant	Effect allele	*β*	Adjusted *p*
*APP*	21:27543049	rs459543	UTR5	c.‐111G > C	G	−157.2	7.91 × 10^−3^

Abbreviations: AD, Alzheimer's disease; Adjusted P, adjusted by age, gender, and *APOE* ε4 status; Aβ42, amyloid‐β 1–42; CSF, cerebrospinal fluid; UTR5, 5′ untranslated region.

## 4 DISCUSSION

In our study, to determine the associations of non‐pathogenic variants of *APP*, *PSEN1*, and *PSEN2* with AD risk, we comprehensively studied *APP*, *PSEN1*, and *PSEN2* genes in a large‐scale Chinese population cohort. The common variant association study revealed that *PSEN2* rs11405 was nominally associated with AD risk. The gene‐based analysis indicated that the *APP* gene reached statistical significance between AD patients and controls. AAO and MMSE association studies demonstrated that none of these variants were linked to AD endophenotypes.

In the 1990s, based on the genetic linkage analysis, *APP*, *PSEN1*, and *PSEN2* were identified as pathogenic genes for AD.^19^, ^20^, ^21^, ^22^ All of these genes increased the production of amyloid‐β (Aβ) or elevated the ratio of amyloid‐β_1‐42_ to amyloid‐β_1‐40_, which subsequently lead to the dominant hypothesis–amyloid hypothesis in AD^23^. The most common genetic causes of AD are pathogenic variants in *PSEN1*, followed by *APP* and *PSEN2* ^24^. Nonetheless, only a few AD patients were caused by variants in these three genes. For example, even in early‐onset Alzheimer's disease patients, less than 5% of them carried the pathogenic variants in *APP*, *PSEN1*, and *PSEN2* ^25^. Additionally, some of the variants have no exact clinical significance, and their associations with AD are needed to be investigated.^26^

Our study revealed that *PSEN2* rs11405 reached nominal significance between AD patients and controls. *PSEN2* is located on chromosome 1q42.13. To date, 63 *PSEN2* variants have been found and only approximately half of them were considered to cause AD^8^. A previous study showed that rare coding variants in *PSEN2* contributed to susceptibility for apparently sporadic late‐onset AD^27^. However, another study found that no variants in *PSEN2* exhibited a significant association with AD risk. Thus, the association of variants in *PSEN2* with AD remains controversial. In this large‐scale Chinese cohort, we firstly identified that a common variant, *PSEN2* rs11405, was nominally correlated with AD risk. Although further studies are needed to replicate this result in AD, our finding indicated that *PSEN2* rs11405 may be involved in the pathogenesis of AD. The role of *PSEN2* rs11405 is needed to be replicated in other larger populations.

Furthermore, we identified that the rare missense variants in *APP* were associated with AD risk. Located on chromosome 21q21.3, the *APP* gene encodes amyloid‐beta precursor protein (APP). APP is a transmembrane protein, and its proteolysis can lead to the production of Aβ protein.^28^ It is reported that a rare coding variant, *APP* p.G322A, increased the risk for late‐onset AD^29^. Nevertheless, two previous studies demonstrated that rare coding variants were not linked to AD risk.^27^, ^30^ Our study firstly found that rare variants in *APP* were linked to AD risk using the gene‐based analysis (SKAT‐O test), indicating the significant role of *APP* in AD pathogenesis.

Additionally, we found that neither common variants in *PSEN1* nor rare variants in *PSEN1* were associated with AD risk. Although a previous study revealed that *PSEN1* p.E318G conferred susceptibility for AD^31^, other studies showed that variants in *PSEN1* were not associated with AD risk.^30^, ^32^, ^33^ Thus, the associations of common *PSEN1* variants with AD remain controversial. Our result further suggested that variants in *PSEN1* may be not correlated with AD risk. Meanwhile, we found that the common variants in *APP*, *PSEN1*, and *PSEN2* were not correlated with AD endophenotypes, including the age of onset and MMSE, suggesting that these variants were not involved in AD development.

In AD endophenotypes association studies, we identified that *APP* rs459543 was nominally correlated with CSF Aβ42 levels. In the Dominantly Inherited Alzheimer's Network (DIAN) observational study, *PSEN1*, *PSEN2*, or *APP* pathogenic variants presented markedly differential Pittsburgh‐Compound‐B PET (PiB‐PET) signal but with similar CSF Aβ42 levels.^34^ Another study revealed that a *PSEN2* haplotype was associated with CSF Aβ42 concentrations.^35^ Our study showed that *APP* rs459543 was suggestively correlated with CSF Aβ42 levels, indicating its potential role in AD biomarker changes. However, only a few AD patients underwent CSF testing and the association disappeared after multiple tests. Therefore, it is necessary to determine whether *APP* rs459543 modulates CSF Aβ42 concentrations in the future.

Although we systematically investigated the role of non‐pathogenic variants of *APP*, *PSEN1*, and *PSEN2* in AD, a few limitations exist. First, our sample size is relatively limited, and the results needed to be replicated in larger sample sizes. Second, we analyzed these genes in the Chinese population, and the results also warrant replication in other populations.

Taken together, we analyzed the non‐pathogenic variants in *APP*, *PSEN1*, and *PSEN2* between AD and controls in a large Chinese cohort. The common variant association test suggested that *PSEN2* rs11405 was nominally linked to AD risk. Rare missense variants in *APP* contributed to the pathogenesis of AD. This study indicated that non‐pathogenic variants in *APP* and *PSEN2* were involved in AD development.

## CONFLICT OF INTEREST

The authors declare that there is no conflict of interest associated with the contents of this article.

## Supporting information


Table S1–S9

## Data Availability

The data that support the findings of this study are available from the corresponding author upon reasonable request.
