# Supplementary material for: The associations of APP , PSEN1 , and PSEN2 genes with Alzheimer's disease: A large case–control study in Chinese population
Source: CNS Neurosci Ther. 2022 Oct 10;29(1):122–8. doi: 10.1111/cns.13987 (PMC9804049; doi:10.1111/cns.13987)
Supplement: Supplementary file 1 — Table S1–S9 [file CNS-29-122-s001.docx]

**Supplementary Table 1. Rare LoF variants in the *APP* gene between AD patients and controls in the SKAT-O test**

| **Classification** | **Gene** | **Location** | **Variant** | **AD (n)** | **Control (n)** |
| --- | --- | --- | --- | --- | --- |
| **Rare LoF variants**  **(MAF<0.01)** | *APP* | 21:27369732 | c.1034-1G>C | 0 | 2 |
| **Allele count / total number of alleles (n/n)** | | | | 0/2308 | 2/4806 |
| **Frequency (%)** | | | | 0 | 0.042 |
| **Adjusted P (SKAT-O)** | | | | 0.34 | |

**Abbreviations:** Adjusted P: adjusted by age, gender, and *APOE* ε4 status; SKAT-O: Sequence Kernel Association Test-Optimal

**Supplementary Table 2. Rare damaging missense variants in the *APP* gene between AD patients and controls in the SKAT-O test**

| **Classification** | **Gene** | **Location** | **Variant** | **AD (n)** | **Control (n)** |
| --- | --- | --- | --- | --- | --- |
| **Rare damaging missense variants**  **(MAF<0.01)** | *APP* | 21:27277356 | c.1943G>A:p.R648Q | 1 | 0 |
|  |  | 21:27284152 | c.1810G>A:p.V604M | 0 | 1 |
|  |  | 21:27284214 | c.1748A>G:p.E583G | 0 | 1 |
|  |  | 21:27327949 | c.1579C>T:p.R527W | 1 | 1 |
|  |  | 21:27328006 | c.1522A>G:p.T508A | 0 | 1 |
|  |  | 21:27372467 | c.896C>G:p.P299R | 1 | 0 |
|  |  | 21:27423508 | c.470C>T:p.T157I | 1 | 0 |
|  |  | 21:27484325 | c.196A>G:p.K66E | 1 | 0 |
| **Allele count / total number of alleles (n/n)** | | | | 5/2308 | 4/4806 |
| **Frequency (%)** | | | | 0.22 | 0.08 |
| **Adjusted P (SKAT-O)** | | | | 0.65 | |

**Abbreviations:** Adjusted P: adjusted by age, gender, and *APOE* ε4 status; SKAT-O: Sequence Kernel Association Test-Optimal

**Supplementary Table 3. Rare damaging variants in the *APP* gene between AD patients and controls in the SKAT-O test**

| **Classification** | **Gene** | **Location** | **Variant** | **AD (n)** | **Control (n)** |
| --- | --- | --- | --- | --- | --- |
| **Rare damaging variants**  **(MAF<0.01)** | *APP* | 21:27369732 | c.1034-1G>C | 0 | 2 |
|  |  | 21:27277356 | c.1943G>A:p.R648Q | 1 | 0 |
|  |  | 21:27284152 | c.1810G>A:p.V604M | 0 | 1 |
|  |  | 21:27284214 | c.1748A>G:p.E583G | 0 | 1 |
|  |  | 21:27327949 | c.1579C>T:p.R527W | 1 | 1 |
|  |  | 21:27328006 | c.1522A>G:p.T508A | 0 | 1 |
|  |  | 21:27372467 | c.896C>G:p.P299R | 1 | 0 |
|  |  | 21:27423508 | c.470C>T:p.T157I | 1 | 0 |
|  |  | 21:27484325 | c.196A>G:p.K66E | 1 | 0 |
| **Allele count / total number of alleles (n/n)** | | | | 5/2308 | 6/4806 |
| **Frequency (%)** | | | | 0.22 | 0.12 |
| **Adjusted P (SKAT-O)** | | | | 0.57 | |

**Abbreviations:** Adjusted P: adjusted by age, gender, and *APOE* ε4 status; SKAT-O: Sequence Kernel Association Test-Optimal

**Supplementary Table 4. Rare missense variants in the *PSEN1* gene between AD patients and controls in the SKAT-O test**

| **Classification** | **Gene** | **Location** | **Variant** | **AD (n)** | **Control (n)** |
| --- | --- | --- | --- | --- | --- |
| **Rare missense variants**  **(MAF<0.01)** | *PSEN1* | 14:73637546 | c.129C>A:p.S43R | 0 | 1 |
|  |  | 14:73640406 | c.471G>T:p.R157S | 0 | 1 |
|  |  | 14:73683843 | c.1139A>G:p.K380R | 1 | 0 |
|  |  | 14:73685962 | c.1369A>G:p.M457V | 1 | 0 |
| **Allele count / total number of alleles (n/n)** | | | | 2/2308 | 2/4806 |
| **Frequency (%)** | | | | 0.087 | 0.042 |
| **Adjusted P (SKAT-O)** | | | | 0.84 | |

**Abbreviations:** Adjusted P: adjusted by age, gender, and *APOE* ε4 status; SKAT-O: Sequence Kernel Association Test-Optimal

**Supplementary Table 5. Rare damaging missense variants in the *PSEN1* gene between AD patients and controls in the SKAT-O test**

| **Classification** | **Gene** | **Location** | **Variant** | **AD (n)** | **Control (n)** |
| --- | --- | --- | --- | --- | --- |
| **Rare damaging missense**  **variants**  **(MAF<0.01)** | *PSEN1* | 14:73640406 | c.471G>T:p.R157S | 0 | 1 |
|  |  | 14:73683843 | c.1139A>G:p.K380R | 1 | 0 |
|  |  | 14:73685962 | c.1369A>G:p.M457V | 1 | 0 |
| **Allele count / total number of alleles (n/n)** | | | | 2/2308 | 1/4806 |
| **Frequency (%)** | | | | 0.087 | 0.021 |
| **Adjusted P (SKAT-O)** | | | | 0.69 | |

**Abbreviations:** Adjusted P: adjusted by age, gender, and *APOE* ε4 status; SKAT-O: Sequence Kernel Association Test-Optimal

**Supplementary Table 6. Rare damaging missense variants in the *PSEN1* gene between AD patients and controls in the SKAT-O test**

| **Classification** | **Gene** | **Location** | **Variant** | **AD (n)** | **Control (n)** |
| --- | --- | --- | --- | --- | --- |
| **Rare damaging**  **variants**  **(MAF<0.01)** | *PSEN1* | 14:73640406 | c.471G>T:p.R157S | 0 | 1 |
|  |  | 14:73683843 | c.1139A>G:p.K380R | 1 | 0 |
|  |  | 14:73685962 | c.1369A>G:p.M457V | 1 | 0 |
| **Allele count / total number of alleles (n/n)** | | | | 2/2308 | 1/4806 |
| **Frequency (%)** | | | | 0.087 | 0.021 |
| **Adjusted P (SKAT-O)** | | | | 0.69 | |

**Abbreviations:** Adjusted P: adjusted by age, gender, and *APOE* ε4 status; SKAT-O: Sequence Kernel Association Test-Optimal

**Supplementary Table 7. Rare missense variants in the *PSEN2* gene between AD patients and controls in the SKAT-O test**

| **Classification** | **Gene** | **Location** | **Variant** | **AD (n)** | **Control (n)** |
| --- | --- | --- | --- | --- | --- |
| **Rare missense variants**  **(MAF<0.01)** | *PSEN2* | 1:227069657 | c.49C>T:p.R17W | 2 | 2 |
|  |  | 1:227069661 | c.53C>T:p.T18M | 0 | 1 |
|  |  | 1:227069678 | c.70G>A:p.E24K | 1 | 1 |
|  |  | 1:227069691 | c.83C>T:p.P28L | 1 | 0 |
|  |  | 1:227069693 | c.85C>T:p.R29C | 0 | 1 |
|  |  | 1:227069708 | c.100G>A:p.G34S | 6 | 29 |
|  |  | 1:227071472 | c.208G>A:p.G70R | 1 | 0 |
|  |  | 1:227073254 | c.372C>G:p.F124L | 0 | 1 |
|  |  | 1:227073264 | c.382A>G:p.T128A | 1 | 0 |
|  |  | 1:227073291 | c.409A>T:p.N137Y | 1 | 1 |
|  |  | 1:227073292 | c.410A>G:p.N137S | 0 | 1 |
|  |  | 1:227073297 | c.415G>A:p.V139M | 0 | 1 |
|  |  | 1:227075798 | c.505C>A:p.H169N | 3 | 3 |
|  |  | 1:227075832 | c.539T>C:p.L180P | 0 | 1 |
|  |  | 1:227076590 | c.627G>C:p.W209C | 0 | 1 |
|  |  | 1:227076603 | c.640G>T:p.V214L | 12 | 19 |
|  |  | 1:227076621 | c.658C>T:p.H220Y | 0 | 1 |
|  |  | 1:227076718 | c.755C>T:p.A252V | 0 | 1 |
|  |  | 1:227077741 | c.793G>A:p.V265M | 1 | 0 |
|  |  | 1:227077760 | c.812A>G:p.K271R | 0 | 1 |
|  |  | 1:227077817 | c.869C>T:p.P290L | 0 | 1 |
|  |  | 1:227079021 | c.929C>T:p.S310F | 0 | 1 |
|  |  | 1:227079535 | c.1062A>C:p.E354D | 0 | 1 |
|  |  | 1:227079539 | c.1066G>A:p.E356K | 0 | 1 |
|  |  | 1:227081729 | c.1094G>C:p.G365A | 1 | 0 |
|  |  | 1:227081774 | c.1139C>T:p.T380M | 0 | 1 |
|  |  | 1:227083237 | c.1304G>A:p.R435Q | 2 | 1 |
| **Allele count / total number of alleles (n/n)** | | | | 32/2308 | 71/4806 |
| **Frequency (%)** | | | | 1.39 | 1.48 |
| **Adjusted P (SKAT-O)** | | | | 0.06 | |

**Abbreviations:** Adjusted P: adjusted by age, gender, and *APOE* ε4 status; SKAT-O: Sequence Kernel Association Test-Optimal

**Supplementary Table 8. Rare damaging missense variants in the *PSEN2* gene between AD patients and controls in the SKAT-O test**

| **Classification** | **Gene** | **Location** | **Variant** | **AD (n)** | **Control (n)** |
| --- | --- | --- | --- | --- | --- |
| **Rare damaging missense variants**  **(MAF<0.01)** | *PSEN2* | 1:227069657 | c.49C>T:p.R17W | 2 | 2 |
|  |  | 1:227069678 | c.70G>A:p.E24K | 1 | 1 |
|  |  | 1:227073254 | c.372C>G:p.F124L | 0 | 1 |
|  |  | 1:227073264 | c.382A>G:p.T128A | 1 | 0 |
|  |  | 1:227073291 | c.409A>T:p.N137Y | 1 | 1 |
|  |  | 1:227073297 | c.415G>A:p.V139M | 0 | 1 |
|  |  | 1:227075798 | c.505C>A:p.H169N | 3 | 3 |
|  |  | 1:227075832 | c.539T>C:p.L180P | 0 | 1 |
|  |  | 1:227076590 | c.627G>C:p.W209C | 0 | 1 |
|  |  | 1:227076603 | c.640G>T:p.V214L | 12 | 19 |
|  |  | 1:227076621 | c.658C>T:p.H220Y | 0 | 1 |
|  |  | 1:227076718 | c.755C>T:p.A252V | 0 | 1 |
|  |  | 1:227077760 | c.812A>G:p.K271R | 0 | 1 |
|  |  | 1:227077817 | c.869C>T:p.P290L | 0 | 1 |
|  |  | 1:227079021 | c.929C>T:p.S310F | 0 | 1 |
|  |  | 1:227079535 | c.1062A>C:p.E354D | 0 | 1 |
|  |  | 1:227079539 | c.1066G>A:p.E356K | 0 | 1 |
|  |  | 1:227081729 | c.1094G>C:p.G365A | 1 | 0 |
|  |  | 1:227081774 | c.1139C>T:p.T380M | 0 | 1 |
| **Allele count / total number of alleles (n/n)** | | | | 21/2308 | 38/4806 |
| **Frequency (%)** | | | | 0.91 | 0.79 |
| **Adjusted P (SKAT-O)** | | | | 0.78 | |

**Abbreviations:** Adjusted P: adjusted by age, gender, and *APOE* ε4 status; SKAT-O: Sequence Kernel Association Test-Optimal

**Supplementary Table 9. Rare damaging variants in the *PSEN2* gene between AD patients and controls in the SKAT-O test**

| **Classification** | **Gene** | **Location** | **Variant** | **AD (n)** | **Control (n)** |
| --- | --- | --- | --- | --- | --- |
| **Rare damaging variants**  **(MAF<0.01)** | *PSEN2* | 1:227069657 | c.49C>T:p.R17W | 2 | 2 |
|  |  | 1:227069678 | c.70G>A:p.E24K | 1 | 1 |
|  |  | 1:227073254 | c.372C>G:p.F124L | 0 | 1 |
|  |  | 1:227073264 | c.382A>G:p.T128A | 1 | 0 |
|  |  | 1:227073291 | c.409A>T:p.N137Y | 1 | 1 |
|  |  | 1:227073297 | c.415G>A:p.V139M | 0 | 1 |
|  |  | 1:227075798 | c.505C>A:p.H169N | 3 | 3 |
|  |  | 1:227075832 | c.539T>C:p.L180P | 0 | 1 |
|  |  | 1:227076590 | c.627G>C:p.W209C | 0 | 1 |
|  |  | 1:227076603 | c.640G>T:p.V214L | 12 | 19 |
|  |  | 1:227076621 | c.658C>T:p.H220Y | 0 | 1 |
|  |  | 1:227076718 | c.755C>T:p.A252V | 0 | 1 |
|  |  | 1:227077760 | c.812A>G:p.K271R | 0 | 1 |
|  |  | 1:227077817 | c.869C>T:p.P290L | 0 | 1 |
|  |  | 1:227079021 | c.929C>T:p.S310F | 0 | 1 |
|  |  | 1:227079535 | c.1062A>C:p.E354D | 0 | 1 |
|  |  | 1:227079539 | c.1066G>A:p.E356K | 0 | 1 |
|  |  | 1:227081729 | c.1094G>C:p.G365A | 1 | 0 |
|  |  | 1:227081774 | c.1139C>T:p.T380M | 0 | 1 |
| **Allele count / total number of alleles (n/n)** | | | | 21/2308 | 38/4806 |
| **Frequency (%)** | | | | 0.91 | 0.79 |
| **Adjusted P (SKAT-O)** | | | | 0.78 | |

**Abbreviations:** Adjusted P: adjusted by age, gender, and *APOE* ε4 status; SKAT-O: Sequence Kernel Association Test-Optimal
